# The Reformed Public House

**Published:** 1923-10

**Authors:** D. C. Dering

**Affiliations:** 76 Teignmouth Road, Cricklewood, N.W.2


### CORRESPONDENCE.

[Correspondence on all subjects is invited, but we cannot be responsible for the opinions expressed by our correspondents, who must give name and address as a guarantee of good faith, but not necessarily for publication. Correspondents are reminded that conciseness greatly facilitates early insertion.]

### The Reformed Public House

*To the Editor of* The Hospital and Health Review.

Sir,—In your recent editorial note on Lady Astor's Bill you remark that " We are very slow at arriving at the reformed public house." The work of the People's Refreshment House Association and other bodies working on " disinterested " lines must, of necessity, be slow and be largely on " pioneer " lines. But we have an excellent example of constructive work in the Carlisle State management area, where a successful experiment, initiated in 1916, has now stood the test of seven years and indicates the direction which licensing reform may properly take.

The area covered by the scheme is the city of Carlisle and about 500 square miles of rural country. The licences have been considerably reduced in number, and of the houses retained many have been rebuilt on " reformed " lines. The managers receive a salary plus a commission on food and non-intoxicants ; no commission is given on the sale of alcoholic drinks, which is thus discouraged. Many reforms have been introduced and the scheme is supported by local opinion and by the testimony of those who have investigated it in the public interest. It has not put the country to any expense, for interest is regularly paid on the capital advances, while two-thirds of the original advances by the Treasury have been repaid.

As the *Daily Chronicle* observed recently, " There is no genuine way of reforming the public house without taking away the incentive to sell alcohol." This is secured under the Carlisle scheme. Why cannot that scheme be applied to all our licensed houses either nationally or by administrative areas ?

D. C. Dering.

76 Teignmouth Road,
Cricklewood, N.W. 2.

